# Efficacy and safety of iguratimod in patients with primary Sjögren’s syndrome: a multicentre randomised controlled trial

**DOI:** 10.1136/rmdopen-2025-006180

**Published:** 2025-12-19

**Authors:** Xiaochan Chen, Ting Zhang, Lifeng Meng, Cheng Xu, Lichun Jiang, Guofang Wang, Tao Hou, Hongzhi Wang, Yongmei Han, Ying Guan, Yingying Wang, Jing Xue

**Affiliations:** 1 Department of Rheumatology, Zhejiang University School of Medicine, Second Affiliated Hospital, Hangzhou, Zhejiang, China; 2 Department of Rheumatology, Zhuji People’s Hospital of Zhejiang Province, Zhuji, Zhejiang, China; 3 Department of Rheumatology, Zhejiang University School of Medicine, Sir Run Run Shaw Hospital, Hangzhou, Zhejiang, China; 4 Department of Rheumatology, First Hospital of Jiaxing, Jiaxing, Zhejiang, China; 5 Department of Rheumatology, Shaoxing Central Hospital, Shaoxing, Zhejiang, China; 6 Department of Rheumatology, Zhejiang University School of Medicine, Second Affiliated Hospital, Changxing, Zhejiang, China

**Keywords:** Hydroxychloroquine, Treatment, Autoimmune Diseases, Clinical Trial

## Abstract

**Objective:**

This multicentre randomised controlled trial aimed to compare the efficacy and safety of iguratimod (IGU) and hydroxychloroquine (HCQ) in patients with active primary Sjögren’s syndrome (pSS).

**Methods:**

Eligible pSS patients were randomised 1:1 to receive IGU (25 mg two times per day) or HCQ (0.2 g two times per day) for 24 weeks. The primary endpoint was the Sjögren’s Syndrome Responder Index-30 (SSRI-30) response rate at week 24. Secondary endpoints included the Sjögren’s Tool for Assessing Response (STAR), European Alliance of Associations for Rheumatology (EULAR) Sjögren’s Syndrome Disease Activity Index (ESSDAI), EULAR Sjögren’s Syndrome Patient Reported Index (ESSPRI) and biomarker changes.

**Results:**

A total of 78 pSS patients were randomised (40 in HCQ group, 38 in IGU group) and 66 patients (35 in HCQ group, 31 in IGU group) completed the 24-week research. SSRI-30 response rate, the primary endpoint, was numerically higher in the IGU group (57.9% vs 40.0%, p=0.114), but with no statistical significance. However, IGU demonstrated significantly higher response rates for key secondary endpoints including STAR (39.5% vs 15.0%, p=0.015) and ESSDAI (21.1% vs 5.0%, p=0.034) response rate. IGU also showed superior IgG reduction (p=0.046). Adverse events were more frequent with IGU (60.6% vs 37.8%) but were mostly mild.

**Conclusion:**

IGU monotherapy demonstrated significant improvements in composite, systemic and serologic outcomes compared with HCQ in active pSS and was well-tolerated. These findings establish IGU as a promising therapeutic option for pSS, particularly in the subset of patients with hyperglobulinaemia.

**Trial registration number:**

NCT04981145.

**WHAT IS ALREADY KNOWN ON THIS TOPIC**

- Primary Sjögren’s syndrome (pSS) lacks approved disease-modifying therapies, with hydroxychloroquine (HCQ) showing limited efficacy.
- Iguratimod (IGU) demonstrates anti-inflammatory and immunosuppressive effects in rheumatoid arthritis, but robust evidence is lacking in pSS.

**WHAT THIS STUDY ADDS**

- This head-to-head randomised controlled trial showed that IGU monotherapy was more effective than HCQ in improving composite, systemic and serological measures in active pSS patients.
- Provides comprehensive safety data supporting IGU’s manageable adverse event profile in the pSS population.

**HOW THIS STUDY MIGHT AFFECT RESEARCH, PRACTICE OR POLICY**

- Evidence from this study supports IGU as a promising therapeutic option for active pSS, especially in patients with hyperglobulinaemia. Further large-scale studies are warranted to confirm its role in the treatment paradigm.

## Introduction

 Primary Sjögren’s syndrome (pSS) is a chronic systemic autoimmune disorder characterised by lymphocytic infiltration of exocrine glands, primarily the salivary and lacrimal glands, leading to xerostomia, keratoconjunctivitis sicca and systemic manifestations involving organs such as the lungs, kidneys and nervous system.^1^ With a global prevalence ranging from 0.044% to 0.078%, pSS predominantly affects middle-aged women and significantly impairs quality of life due to its chronic and progressive nature.^2^ The pathogenesis of pSS is multifactorial, involving genetic predisposition, environmental triggers and dysregulated immune responses. Central to its pathophysiology is B-cell hyperactivity, which drives the production of autoantibodies such as anti-SSA/Ro and anti-SSB/La, hyperglobulinaemia and elevated levels of proinflammatory cytokines such as interleukin (IL)−17, and B-cell activating factor (BAFF).^3 4^ These immune aberrations drive glandular destruction, systemic inflammation and the development of extraglandular complications, which are associated with increased morbidity and mortality.

Current therapeutic strategies for pSS primarily focus on symptomatic management of sicca symptoms and immunosuppression for systemic manifestations. Traditional immunosuppressants, such as methotrexate, azathioprine and mycophenolate, are commonly used off-label in pSS. However, none of the immunomodulatory drugs have demonstrated definitive efficacy.^5 6^ Hydroxychloroquine (HCQ), an antimalarial drug with immunomodulatory properties, has shown modest efficacy in improving sicca symptoms and some laboratory parameters such as erythrocyte sedimentation rate (ESR) and immunoglobulin (Ig)A in open-label studies.^7 8^ Nevertheless, HCQ did not significantly improve glandular function or reduce systemic disease activity.^9 10^ This situation highlights the need for more effective therapies.

Biologic therapies targeting B cells, such as rituximab (RTX), a monoclonal antibody against CD20, have shown promise in pSS. RTX depletes B cells and reduces autoantibody production, potentially improving glandular function and systemic symptoms in some patients. Randomised controlled trials (RCTs) demonstrated that RTX significantly improved sicca symptoms, salivary flow rates, salivary gland ultrasound scores and several laboratory parameters in pSS patients.^11 12^ However, the high cost, risk of infusion reactions and potential for severe infections limit the widespread use of RTX in pSS.

Given the limitations of existing therapies, there is an urgent need for targeted treatments that modulate B-cell activity and inflammatory pathways with improved safety. Iguratimod (IGU), a small-molecule immunosuppressant approved for rheumatoid arthritis in Asia, has emerged as a potential therapeutic option for pSS. IGU exerts its effects through multiple mechanisms: it inhibits the production of proinflammatory cytokines such as tumour necrosis factor-α, IL-6 and IL-17, suppresses B-cell differentiation and activation and reduces Ig production.^13^ Preclinical studies indicate that IGU significantly decreases levels of BAFF and IL-17,^14 15^ key cytokines in pSS pathogenesis, and inhibits terminal B-cell differentiation in vitro.^16^ These mechanisms suggest that IGU may effectively target the underlying immune dysregulation in pSS.

Preliminary clinical evidence supports IGU’s efficacy in pSS. An open-label pilot study reported that IGU treatment significantly reduced disease activity scores (European Alliance of Associations for Rheumatology (EULAR) Sjögren’s Syndrome Disease Activity Index, ESSDAI) and normalised IgG and rheumatoid factor (RF) levels in pSS patients.^17^ Another RCT demonstrated that IGU improved sicca symptoms and patient-reported outcomes (EULAR Sjögren’s Syndrome Patient Reported Index, ESSPRI), while reducing BAFF levels and plasma cell percentages.^18^ Furthermore, a meta-analysis of 19 RCTs found that IGU effectively reduced ESSPRI and ESSDAI scores, improved Schirmer’s test results and decreased systemic inflammation (ESR and RF levels).^19^ These findings supported the 2023 Chinese recommendations that endorsed IGU as a therapeutic option for pSS.^20^ However, robust comparative data against standard therapies like HCQ are lacking, and the long-term safety and efficacy of IGU in pSS remain incompletely defined.

This multicentre RCT aims to evaluate the efficacy and safety of IGU monotherapy versus HCQ in patients with active pSS. By addressing gaps in the evidence for targeted B-cell modulation, this study seeks to provide a foundation for developing more effective and safe treatment strategies.

## Materials and methods

### Study design

This open-label, parallel-group superiority RCT was conducted across six centres in China: Second Affiliated Hospital, Zhejiang University School of Medicine, Sir Run Run Shaw Hospital Zhejiang University School of Medicine, The First Hospital of Jiaxing, Zhuji People’s Hospital, Changxing People’s Hospital and Shaoxing Central Hospital. The study protocol was approved by institutional ethics committees at each centre and registered at ClinicalTrials.gov (NCT04981145). All participants provided written informed consent.

### Participants and treatment protocol

Inclusion criteria: (1) aged 18–70 years; (2) fulfilment of the 2016 American College of Rheumatology/EULAR classification criteria for pSS; (3) seropositivity for anti-Ro-60/SSA antibodies; (4) active oral or ocular dryness symptoms; (5) associated with hyperglobulinaemia (Ig≥16 g/L); (6) no glucocorticoids, immunosuppressants or biologics within 4 weeks prior to screening. Key exclusion criteria: (1) pregnancy, lactation or inadequate contraception; (2) comorbid autoimmune diseases (systemic lupus erythematosus, rheumatoid arthritis, systemic sclerosis, etc); (3) comorbid malignancies; (4) severe organ dysfunction (hepatic, renal, pulmonary, etc), requiring glucocorticoid therapy; (5) history of hypersensitivity to IGU or HCQ.

Participants were randomised 1:1 using a stratified randomisation method based on study centres, implemented via a centralised, third-party interactive web-response system. Eligible participants received either IGU (25 mg two times per day) or HCQ (0.2 g two times per day) for 24 weeks. Concomitant use of glucocorticoids, other immunosuppressants, biologics or other drugs potentially affecting efficacy evaluation was prohibited during the study period.

### Follow-up and data collection

Participants attended visits at week 0 (baseline), 2, 6, 10, 16 and 24. Demographic data (age, sex, disease duration), antinuclear antibody profiles and comorbidities were recorded at baseline. ESSDAI and ESSPRI scores, Schirmer’s test, unstimulated salivary flow rate (USFR) and lymphocyte subsets were evaluated at baseline and week 24. Complete blood count, urinalysis, liver and kidney function, immunoglobulins, complements, RF, ESR and CRP were collected at each visit. Adverse events (AEs) were recorded at each visit simultaneously.

### Endpoints

The primary endpoint was the Sjögren’s Syndrome Responder Index-30 (SSRI-30) response rate at week 24.^21^ This composite endpoint requires ≥30% improvement in at least two of five core measures: fatigue, oral and ocular dryness visual analogue scale scores, USFR and ESR.

The key secondary endpoint was the Sjögren’s Tool for Assessing Response (STAR) response rate at week 24, an internationally endorsed composite score developed in 2022 via Delphi consensus.^22^ STAR integrates five core domains: systemic activity (clinESSDAI), patient symptoms (ESSPRI), lacrimal gland function (Schirmer’s test/ocular staining score), salivary gland function (USFR/ultrasound), and biological parameters (IgG/RF). It employs a weighted scoring system (systemic activity and symptoms: 3 points each; other domains: 1 point each), with a total score ≥5 defining response. Other secondary endpoints included changes in ESSDAI score (response defined as decrease ≥3), ESSPRI score (response defined as decrease ≥1 point or ≥15%), USFR, Schirmer’s test, immunoglobulins, RF, ESR and lymphocyte subsets over 24 weeks.

### Statistical analysis

Sample size was calculated using PASS V.15.0. Assuming a 17.6% SSRI-30 response rate for HCQ^9^ versus 50% for IGU (α=0.05, power=80%, dropout=15%), 78 participants were required. The main efficacy analysis was performed according to the intention-to-treat (ITT) principle, which included all randomised patients. Missing data for the endpoints were handled using the ‘treatment failure’ rule, where patients with missing outcomes were considered non-responders. A per-protocol (PP) analysis was conducted as a sensitivity analysis to assess the robustness of the primary findings. Data were analysed using SPSS software V.22.0 (IBM, Armonk, New York). Continuous variables were compared via t-tests or Mann-Whitney U tests; categorical variables via χ^2^ or Fisher’s exact tests. Linear regression analysis was used to evaluate the influence of treatment on changes in scores and biomarkers. Two-sided p values <0.05 were considered statistically significant.

## Results

### Baseline characteristics

**Figure 1:**
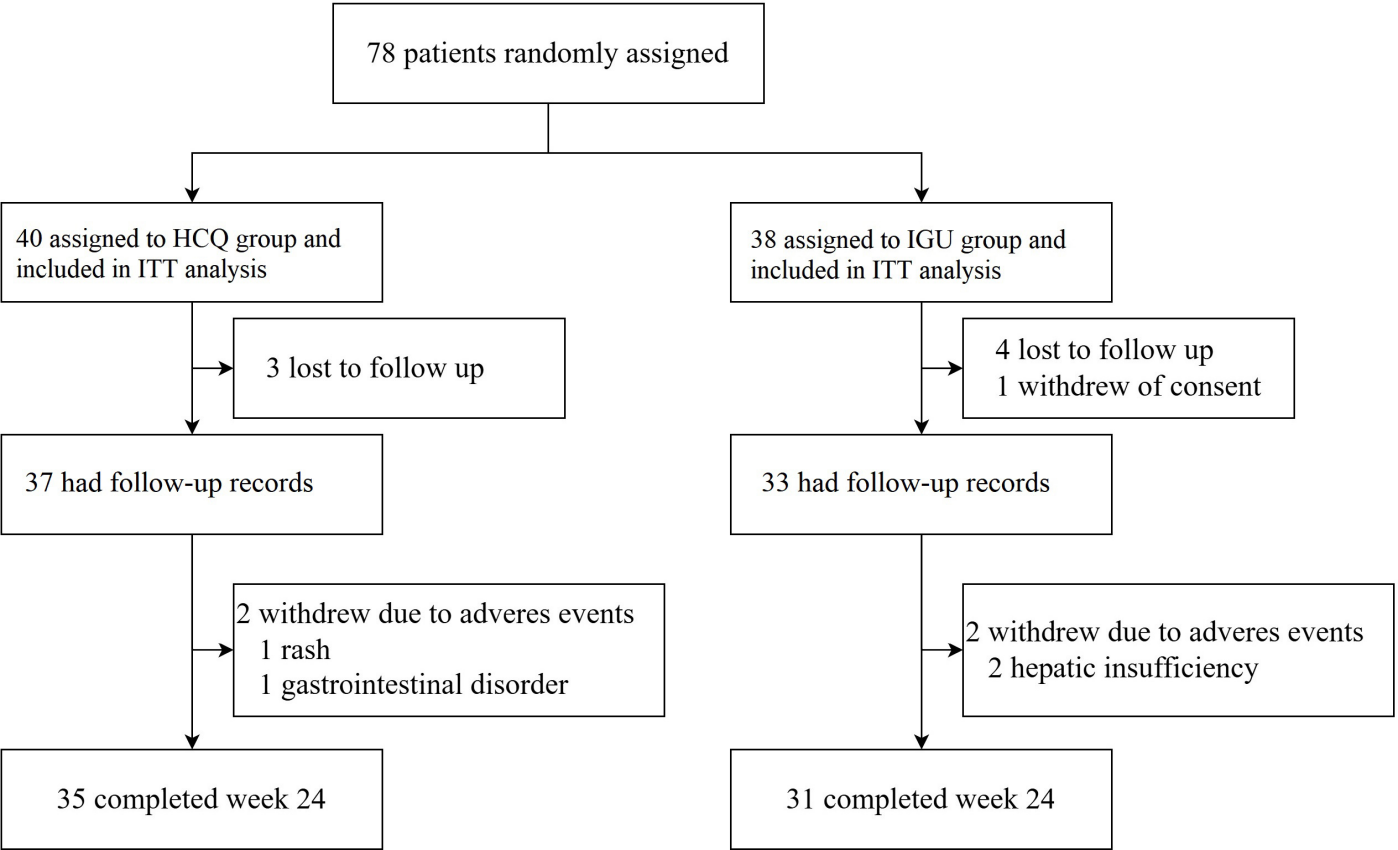
Trial profile. HCQ, hydroxychloroquine; IGU, iguratimod; ITT, intention-to-treat.

A total of 78 eligible patients with active pSS were randomised: 40 to HCQ and 38 to IGU. As shown in figure 1, eight patients (three in HCQ group, five in IGU group) had no follow-up data due to loss to follow-up or withdrawal of consent after randomisation. Among the 70 patients who received study drug therapy, 2 patients in each group discontinued treatment prematurely due to AEs. Consequently, 35 patients in the HCQ group (87.5% of HCQ randomised) and 31 patients in the IGU group (81.6% of IGU randomised) completed the 24-week course.

Demographic and clinical characteristics at baseline were largely comparable between groups (table 1). Both cohorts exhibited the typical pSS phenotype: predominantly female (HCQ: 97.5% (39/40), IGU: 97.4% (37/38); p=1.000) and middle-aged (HCQ: 45.3±11.3 years, IGU: 48.7±11.6 years; p=0.187). Median disease duration was 3.0 years in the HCQ group and 4.5 years in the IGU group (p=0.263). Serologically, all patients were anti-Ro/SSA positive, while anti-La/SSB positivity was numerically higher in the IGU group (57.9% (22/38) vs 37.5% (15/40); p=0.071), though not statistically significant. Systemic manifestations occurred at comparable frequencies, with haematological involvement being most common (HCQ: 27.5% (11/40), IGU: 21.1% (8/38)). Critically, baseline disease activity indices (ESSDAI, ESSPRI), serum inflammatory markers (IgG, ESR, CRP, RF) and glandular function measures (USFR, Schirmer’s test) showed no statistically significant differences.

**Table 1:** Baseline demographic and disease characteristics of the patients in two groups

	HCQ (n=40)	IGU (n=38)	P values
Female	39 (97.5%)	37 (97.4%)	1.000
Age (years)	45.3±11.3	48.7±11.6	0.187
Disease duration (years)	3.0 (0.7–5.0)	4.5 (1.0–8.0)	0.263
Anti-Ro-60/SSA antibodies	40 (100%)	38 (100.0%)	1.000
Anti-Ro-52 antibodies	32 (80.0%)	31 (81.6%)	0.860
Anti-La/SSB antibodies	15 (37.5%)	22 (57.9%)	0.071
Organ involvement			
Glandular	3 (7.5%)	4 (10.5%)	0.640
Articular	7 (17.5%)	5 (13.2%)	0.595
Cutaneous	3 (7.5%)	7 (18.4%)	0.270
Pulmonary	4 (10.0%)	3 (7.9%)	1.000
Haematological	11 (27.5%)	8 (21.1%)	0.507
Renal	2 (5.0%)	3 (7.9%)	0.953
Peripheral nervous system	1 (2.5%)	2 (5.3%)	0.964
ESSDAI score	2.0 (1.0–4.5)	2.5 (2.0–4.0)	0.149
ESSPRI score	3.0 (2.0–3.4)	3.0 (2.3–4.0)	0.186
Ocular dryness (VAS,1–10)	3.0 (2.0–4.0)	4.0 (2.0–5.0)	0.526
Oral dryness (VAS,1–10)	4.0 (3.0–6.0)	5.0 (4.0–6.0)	0.142
IgG (g/L)	20.2 (17.9–23.4)	22.2 (19.2–24.9)	0.116
C3 (g/L)	0.94 (0.85–1.12)	1.04 (0.95–1.18)	0.061
C4 (g/L)	0.209 (0.170–0.246)	0.215 (0.190–0.280)	0.224
ESR (mm/h)	24 (14–40)	27 (21–50)	0.148
CRP (mg/L)	1.1 (0.5–2.1)	1.3 (0.6–3.5)	0.484
RF	21.0 (9.4–51.2)	39.0 (22.2–74.1)	0.166
USFR (mL/15 min)	0.7 (0.2–1.9)	0.3 (0.1–0.7)	0.119
Schirmer’s test (mm/5 min)	4.0 (2.8–9.0)	3.5 (0.5–6.0)	0.087

Data are presented as number (%), mean±SD deviation or median (interquartile range).

C3/C4, complement 3/4; CRP, C reactive protein; ESR, erythrocyte sedimentation rate; ESSDAI, EULAR Sjogren’s Syndrome Disease Activity Index; ESSPRI, EULAR Sjögren’s Syndrome Patient Reported Index; HCQ, hydroxychloroquine; IgG, immunoglobulin G; IGU, iguratimod; RF, rheumatoid factor; USFR, unstimulated salivary flow rate; VAS, visual analogue scale.

### Efficacy outcomes

**Figure 2:**
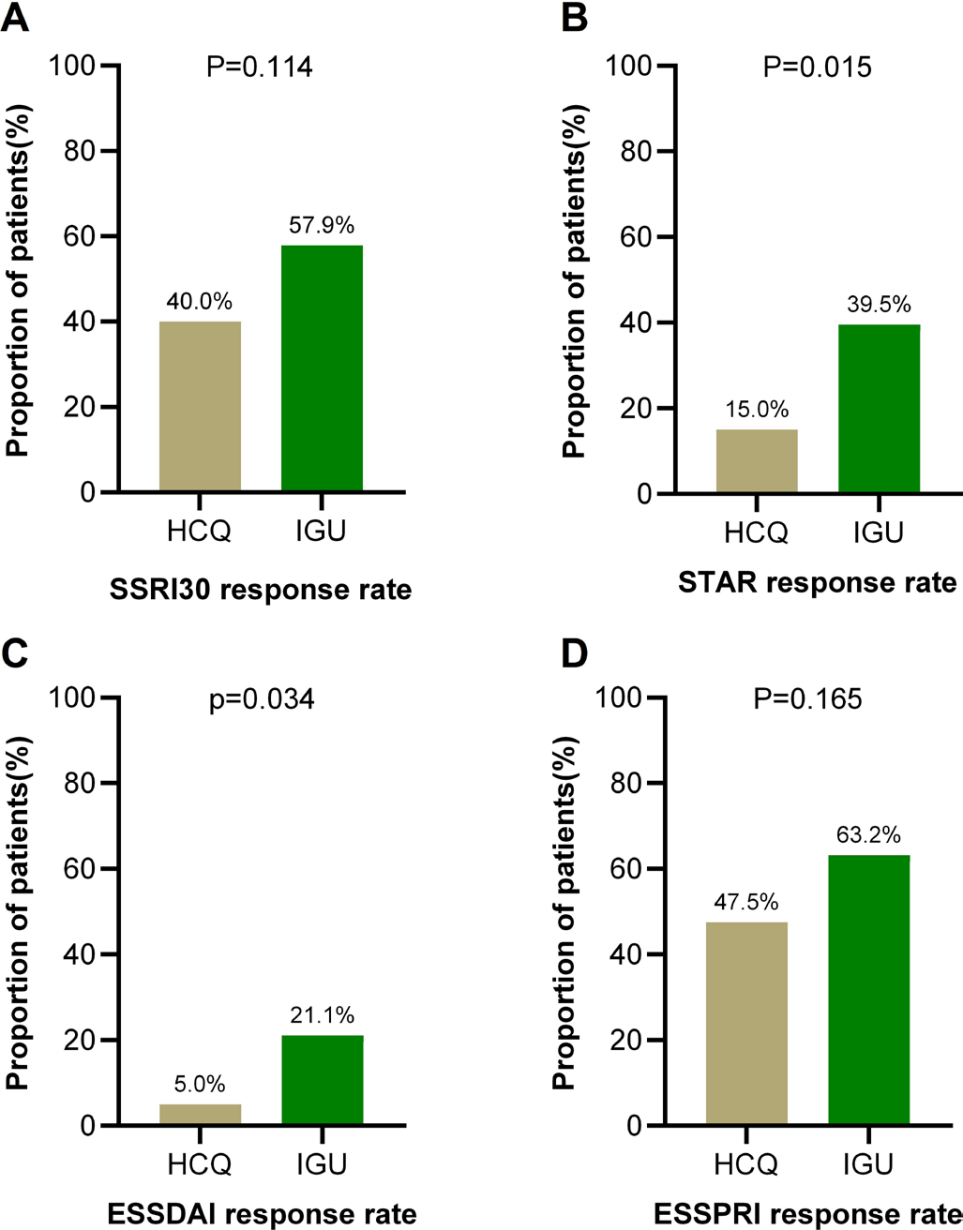
Response rates of SSRI-30 (primary endpoint) and other important efficacy indicators (secondary endpoints) based on the intention-to-treat analysis at week 24. ESSDAI, EULAR Sjögren’s Syndrome Disease Activity Index; ESSPRI, EULAR Sjögren’s Syndrome Patient Reported Index; HCQ, hydroxychloroquine; IGU, iguratimod; ITT, intention-to-treat; SSRI-30, Sjögren’s Syndrome Responder Index-30; STAR, Sjögren’s Tool for Assessing Response.

In the primary endpoint analysis based on the ITT principle, the SSRI-30 response rate at week 24 was numerically higher in the IGU group (57.9%, 22/38) than in the HCQ group (40.0%, 16/40); however, this difference did not reach statistical significance (p=0.114) (figure 2A). For the key secondary endpoints in the ITT population (figure 2B–D), significant differences favouring IGU were observed in the STAR response rate (39.5% vs 15.0%, p=0.015) and the ESSDAI response rate (21.1% vs 5.0%, p=0.034). The difference in ESSPRI response rate, while favouring IGU (63.2% vs 47.5%), was not statistically significant (p=0.165).

A subsequent PP sensitivity analysis, which included only patients who completed the 24-week course (HCQ n=35; IGU n=31), yielded stronger effect estimates. In this population, the SSRI-30 response rate was significantly higher in the IGU group (71.0% vs 45.7%, p=0.038). Similarly, the differences in key secondary endpoints—STAR (48.4% vs 17.1%, p=0.007), ESSDAI (25.8% vs 5.7%, p=0.037) and ESSPRI (77.4% vs 54.3%, p=0.049)—were statistically significant and consistently favoured IGU (online supplemental figure 1).

**Figure 3:**
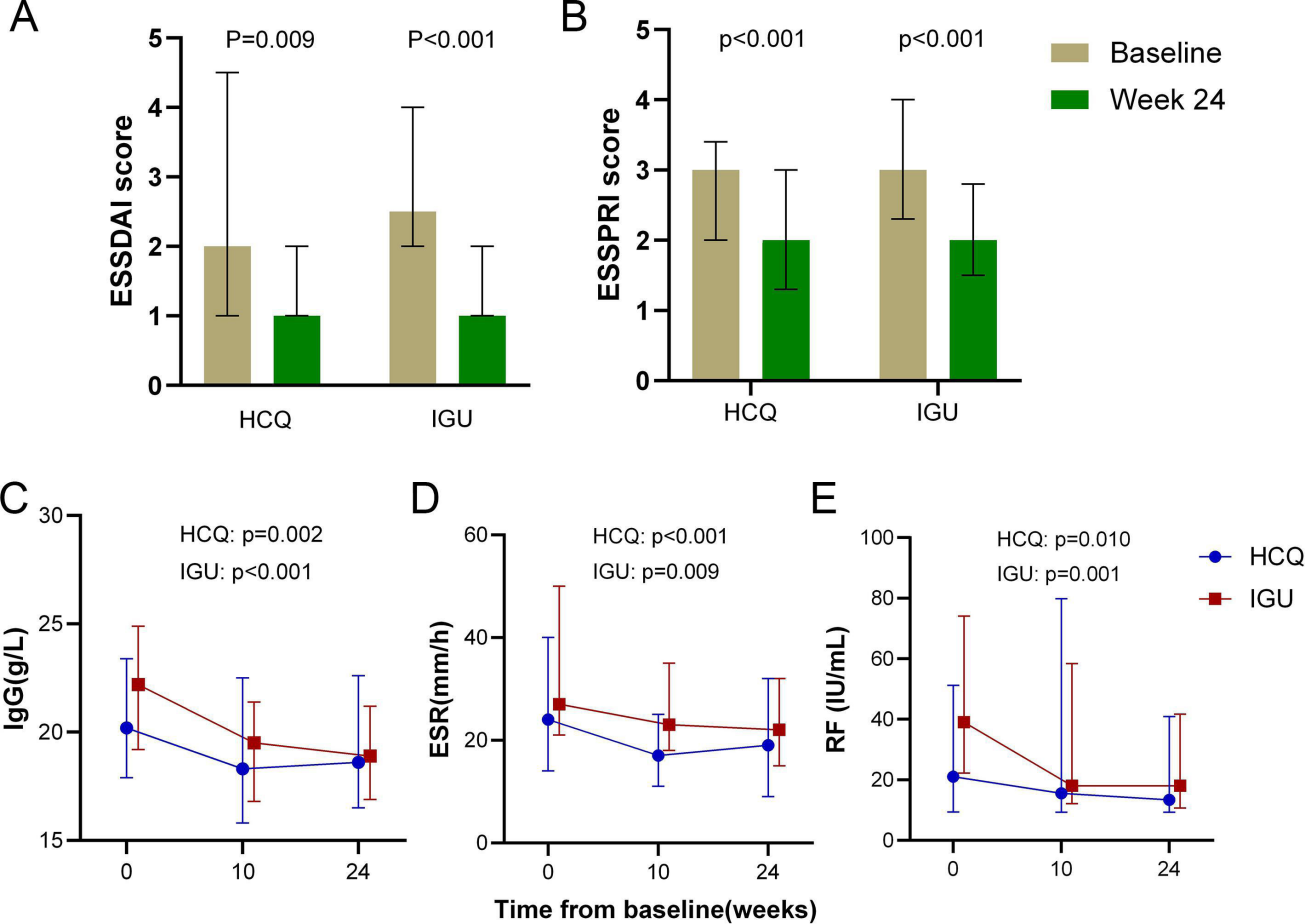
Change of ESSDAI score, ESSPRI score and serum biomarkers over 24 weeks. Error bars represent interquartile range. ESR, erythrocyte sedimentation rate; ESSDAI, EULAR Sjögren’s Syndrome Disease Activity Index; ESSPRI, EULAR Sjögren’s Syndrome Patient Reported Index; HCQ, hydroxychloroquine; IgG, immunoglobulin G; IGU, iguratimod; RF, rheumatoid factor.

Both therapies significantly reduced systemic and symptomatic burden over 24 weeks. ESSDAI scores decreased from baseline in both groups, with statistical significance (p<0.05) for within-group changes (figure 3A). However, between-group differences in absolute change of ESSDAI scores were non-significant after baseline adjustment (linear regression coefficient B (95% CI) −0.42 (−1.09 to 0.24); p=0.208; table 2). ESSPRI scores also improved significantly within groups (p<0.05; figure 3B), with a non-significant trend favouring IGU in magnitude of change (B (95% CI) −0.35 (−0.72 to 0.02); p=0.060).

**Table 2:** Key efficacy and biomarker data in linear regression analysis at week 24

	Baseline		Week 24		B (95% CI)	P values
	HCQ (n=40)	IGU (n=38)	HCQ (n=35)	IGU (n=31)		
ESSDAI score	2.0 (1.0–4.5)	2.5 (2.0–4.0)	1.0 (1.0–2.0)	1.0 (1.0–2.0)	−0.42 (−1.09 to 0.24)	0.208
ESSPRI score	3.0 (2.0–3.4)	3.0 (2.3–4.0)	2.0 (1.3–3.0)	2.0 (1.5–2.8)	−0.35 (−0.72 to 0.02)	0.060
Serum biomarkers						
IgG (g/L)	20.2 (17.9–23.4)	22.2 (19.2–24.9)	18.6 (16.5–22.6)	18.9 (16.9–21.2)	−1.26 (−2.49 to 0.02)	0.046*
ESR (mm/h)	24 (14–40)	27 (21–50)	19 (9–32)	22 (15–32)	−0.10 (−4.48 to 4.68)	0.965
RF (IU/mL)	21.0 (9.4–51.2)	39.0 (22.2–74.1)	13.4 (9.3–40.9)	18.0 (10.7–41.7)	−4.35 (−20.71 to 12.01)	0.597
Gland function						
USFR (mL/15 min)	0.7 (0.2–1.9)	0.3 (0.1–0.7)	0.65 (0.2–1.5)	0.5 (0.2–1.2)	−0.19 (−0.63 to 0.25)	0.380
Schirmer’s test (mm/5 min)	4.0 (2.8–9.0)	3.5 (0.5–6.0)	4.0 (2.0–7.0)	3.0 (1.3–4.3)	−0.38 (−2.17 to 2.94)	0.763
Immune cells	(n=21)	(n=19)	(n=17)	(n=18)		
CD4+T cell (number/μL)	545 (372–641)	460 (370–556)	478 (363–628)	459 (275–662)	−36.7 (−186.1 to 112.6)	0.617
CD8+T cell (number/μL)	421 (259–494)	385 (287–510)	425 (268–525)	433 (279–472)	−50.6 (−155.0 to 53.8)	0.329
CD19+B cell (number/μL)	186 (132–278)	247 (141–310)	175 (114–217)	169 (134–214)	−5.72 (−83.40 to 71.96)	0.881
NK cell (number/μL)	138 (112–270)	160 (90–300)	199 (110–331)	168 (84–273)	−47.0 (−135.1 to 41.1)	0.283

Data are presented as median (interquartile range). Linear regression analysis was constructed applying absolute change in activity scores or biomarker levels as the dependent variable and treatment category (HCQ or IGU) as well as baseline values as two independent variables.

* p<0.05.

B, regression coefficient of treatment category; ESR, erythrocyte sedimentation rate; ESSDAI, EULAR Sjogren’s Syndrome Disease Activity Index; ESSPRI, EULAR Sjögren's Syndrome Patient Reported Index; HCQ, hydroxychloroquine; IgG, immunoglobulin G; IGU, iguratimod; RF, rheumatoid factor; USFR, unstimulated salivary flow rate.

Significant reductions in serum IgG, ESR and RF levels occurred progressively from baseline through weeks 10 and 24 in both arms (p<0.05 for temporal trends; figure 3C–E). After baseline adjustment, IGU demonstrated significantly greater reductions in IgG compared with HCQ (B (95% CI) −1.26 (−2.49 to 0.02); p=0.046; table 2). No significant between-group differences were observed for ESR (B (95% CI) −0.10 (−4.48 to 4.68); p=0.965) or RF (B (95% CI) −4.35 (−20.71 to 12.01); p=0.597). Neither group showed significant improvements in USFR or Schirmer’s test, and no between-group differences were detected. Lymphocyte subset analyses (CD4+T cells, CD8+T cells, CD19+B cells and NK cells) revealed no significant treatment-related alterations (table 2).

### Safety profile

Safety analysis was performed in all patients who received study drug therapy and had at least one follow-up record. AEs were reported in 60.6% (20/33) of IGU recipients versus 37.8% (14/37) of HCQ-treated patients (table 3). Discontinuation rates due to AEs were comparable (HCQ: 5.4% (2/37), IGU: 6.1% (2/33)). Two IGU-treated patients withdrew due to hepatic abnormalities and two HCQ recipients discontinued (one for rash, one for gastrointestinal intolerance). All non-discontinuation AEs resolved with supportive care. No severe AEs or deaths occurred.

**Table 3:** Summary of adverse events

Patients with events, n	HCQ (n=37)	IGU (n=33)	P values
AEs	14 (37.8%)	20 (60.6%)	0.057
Discontinued study due to AEs	2 (5.4%)	2 (6.1%)	1.000
Leucopenia	2 (5.4%)	4 (12.1%)	0.411
Anaemia	1 (2.7%)	1 (3.0%)	1.000
Thrombocytopeniaa	2 (5.4%)	2 (6.1%)	1.000
Upper respiratory tract infections	3 (8.1%)	2 (6.1%)	1.000
Urinary tract infections	1 (2.7%)	0	1.000
Transaminase elevations	1 (2.7%)	7 (21.2%)	0.022*
Serum creatinine increases	3 (8.1%)	5 (15.2%)	0.462
Gastrointestinal disorders	1 (2.7%)	2 (6.1%)	0.599
Hypogeusia	0	1 (3.0%)	0.471
Rash	1 (2.7%)	0	1.000
SAEs	0	0	1.000

Data are numbers of patients, rather than numbers of events, some patients might have had more than one event.

* p<0.05.

AEs, adverse events; HCQ, hydroxychloroquine; IGU, iguratimod; SAEs, severe adverse events.

The most frequent AEs are summarised in table 3. Hepatic events were most common with IGU, with transaminase elevations in 21.2% (7/33) versus 2.7% (1/37) with HCQ. Renal safety monitoring revealed asymptomatic serum creatinine elevation in 15.2% (5/33) of patients in IGU group versus 8.1% (3/37) in HCQ group. Haematological abnormalities included leucopenia (IGU: 12.1% (4/33) vs HCQ: 5.4% (2/37)), thrombocytopenia (two cases per group) and anaemia (one case per group). Gastrointestinal intolerance affected 6.1% (2/33) of IGU recipients versus 2.7% (1/37) of HCQ-treated patients. Infection rates were similar (HCQ: 10.8% (4/37), IGU: 6.1% (2/33)), primarily upper respiratory tract infections. Other notable AEs included one case of transient hypogeusia with IGU and a single rash in HCQ group.

## Discussion

This multicentre RCT provides important evidence on the efficacy and safety of IGU compared with HCQ in patients with active pSS. While the primary ITT analysis of the SSRI-30 response rate did not show a statistically significant difference, the study demonstrated consistent and significant benefits of IGU across multiple key secondary endpoints, including the comprehensive STAR composite score and the physician-assessed ESSDAI. The significantly higher STAR (39.5% vs 15.0%, p=0.015) and ESSDAI (21.1% vs 5.0%, p=0.034) response rates with IGU in the ITT population highlight its capacity for systemic disease control. Moreover, a prespecified sensitivity analysis in the PP population reinforced the robustness of the treatment effect, showing statistically significant improvements for IGU in SSRI-30, STAR, ESSDAI and ESSPRI.

These findings assume particular importance in the context of current research priorities in pSS therapeutics, which emphasise targeted B-cell modulation, development of validated composite endpoints and identification of predictive biomarkers. The field is increasingly shifting from symptom palliation to disease modification, with emerging biologics targeting BAFF/APRIL pathways (eg, ianalumab) and costimulatory molecules (eg, dazodalibep), showing promise in early-phase trials.^23 24^

Although RTX, an off-label therapy for pSS with severe systemic involvement, has demonstrated favourable efficacy in several clinical trials,^25^ two pivotal RCTs (TEARS and TRACTISS) failed to meet their primary endpoints.^26 27^ This discrepancy likely stems from the substantial heterogeneity within the pSS patient population and suboptimal selection of primary endpoints that inadequately captured the multidimensional nature of clinical response. These challenges underscore the critical need for validated composite endpoints capable of comprehensively assessing treatment efficacy.

Given the difficulty in defining disease activity in pSS and the slow progression of the condition, selecting appropriate endpoints for clinical trials poses significant challenges. The selection of SSRI-30 and STAR as endpoints represents a methodological strength. SSRI-30, derived from the TEARS trial, demonstrated significant discriminative capacity in RTX trials for pSS.^21^ STAR, developed via international expert consensus,^22^ integrates clinician-assessed systemic activity, patient symptoms, glandular function and biological parameters, addressing historical challenges in pSS trial design. SSRI-30 is a patient-centric composite that primarily captures improvements in dryness, fatigue and ESR but does not directly weight systemic organ involvement. This characteristic may explain the divergence between the SSRI-30 result and the significant benefits observed in endpoints that specifically assess systemic disease activity (ESSDAI) and the multidimensional, weighted composite (STAR). The significant findings on STAR and ESSDAI response rate are clinically meaningful as they reflect IGU’s capacity to mitigate systemic manifestations—a key therapeutic goal. These results align with IGU’s known dual suppression of proinflammatory cytokines (such as IL-17, BAFF) and B-cell hyperactivity, which are central pathogenic drivers in pSS.^14 18^

It should be noted that, although the binary ESSDAI response rate reached statistical significance between the two groups, the difference in absolute changes in ESSDAI scores did not. This discrepancy can be explained by the nature of our study population and the endpoints used. Our cohort consisted of patients with relatively low baseline ESSDAI scores, indicative of mild systemic involvement. This floor effect inherently limited the potential for substantial absolute score reductions across both groups. In this context, the binary responder analysis, which defines a clinically meaningful improvement (≥3-point decrease), proved to be a more sensitive measure of a treatment effect. It successfully identified a subset of patients in the IGU group who achieved this important threshold, a difference that might be diluted in an analysis of mean changes, especially in a trial of small sample size.

Additionally, our study protocol was finalised in 2021, while the STAR composite endpoint was subsequently developed and published in 2022.^22^ Although STAR was not prespecified in our original trial design, we were able to incorporate this novel endpoint post hoc as our study had systematically collected all required data components (including clinESSDAI, ESSPRI, Schirmer’s test, USFR and relevant serological markers) according to the standardised assessment schedule. This retrospective inclusion strengthens our secondary endpoint analysis by applying the most current, internationally validated outcome measure.

A recent study has compared the effect of IGU and HCQ combined with prednisone (≤10 mg/day) in the treatment of pSS. It demonstrated that both therapies reduced the disease activity (assessed by ESSDAI and ESSPRI), while IGU was superior to HCQ in reducing IgG levels.^28^ Our analyses came to a similar conclusion that IGU treatment resulted in a significantly greater reduction in serum IgG levels compared with HCQ (−1.26 g/L, p=0.046). This finding is consistent with IGU’s established mechanism of suppressing B-cell differentiation and immunoglobulin production.^13^ Notably, although not statistically significant, a numerical difference existed between groups in median IgG level at baseline (22.2 g/L in IGU vs 20.2 g/L in HCQ), which may have influenced the outcome regarding IgG reduction. Nevertheless, the consistent direction of benefit across multiple efficacy measures supports a true treatment effect. This finding holds particular clinical relevance given the established association between hyperglobulinemia and systemic disease manifestations in pSS.^29^

The lack of significant improvement in glandular function (Schirmer’s test, USFR) in both arms aligns with previous trials of immunosuppressants, including belimumab and leflunomide,^30 31^ likely reflecting the challenge of reversing established glandular destruction and fibrosis. This underscores the importance of early intervention before irreversible damage occurs. The absence of significant changes in lymphocyte subsets provides additional mechanistic insight. Unlike B-cell depleting therapies (eg, RTX), IGU primarily modulates B-cell activation and terminal differentiation without causing profound lymphopenia.^13^ This selective immunomodulation may explain its favourable safety profile despite higher overall AE incidence (60.6% vs. 37.8%). The most frequent IGU-associated AEs—transaminase elevations (21.2%) and asymptomatic creatinine increases (15.2%)—were manageable without discontinuation in most cases. This contrasts with RTX’s infusion reactions and infection risks, positioning IGU as a viable option for long-term management.

Our study enrolled a specific cohort of pSS patients characterised by seropositivity for anti-Ro/SSA antibodies, active symptomatic burden and associated hyperglobulinaemia (IgG≥16 g/L). This design ensured the inclusion of a homogeneous population with immunologically active disease, thereby enhancing the internal validity for detecting a treatment signal. However, the generalisability of our findings to the broader pSS population, including seronegative patients or those without hyperglobulinaemia, requires further investigation. Future studies in more diverse, real-world cohorts are necessary to confirm the external validity and applicability of these results.

This study introduces several innovations: first, it represents the inaugural head-to-head comparison between monotherapy of IGU and HCQ, providing direct evidence for therapeutic superiority. Second, it incorporates the novel STAR composite endpoint shortly after its international validation. Third, it elucidates differential biomarker responses illuminating IGU’s mechanism of action in pSS.

These advances must be interpreted alongside limitations. The open-label design introduces potential observer bias, though objective biomarkers (IgG, ESR) corroborated clinical findings. The moderate sample size limited the power for subgroup analyses and may have contributed to the non-significant result for the primary ITT analysis. The 24-week duration of this trial is another critical consideration. HCQ is known to have a slow onset of action, often requiring 6–12 months to achieve its full immunomodulatory effect.^7 9^ In contrast, the pharmacological action of IGU, which directly suppresses pro-inflammatory cytokine production and B-cell differentiation, may lead to a more rapid clinical response.^13^ This difference in time-to-effect could have favoured IGU in the context of our 24-week trial. A longer term follow-up study would be valuable to determine if the treatment effects converge or diverge over time. Additionally, the absence of salivary gland ultrasonography or histology limited assessment of structural changes.

Future research should prioritise long-term extension studies to assess IGU’s sustained efficacy and durability, combination trials with B-cell depleting agents (eg, IGU+RTX) to leverage synergistic mechanisms, and biomarker validation studies to identify predictors of treatment response. Health economic analyses comparing IGU with biologics will further inform clinical implementation.

## Conclusion

In this trial, IGU demonstrated significant benefits over HCQ in improving composite (STAR), systemic (ESSDAI) and serological (IgG) outcomes in patients with active pSS, with a manageable safety profile. These results provide robust evidence supporting IGU as a promising disease-modifying therapy and a viable candidate for the treatment of active pSS, particularly in patients with features of B-cell hyperactivity. Future larger scale and longer duration studies are now warranted to confirm these findings and to definitively establish IGU’s role in the pSS treatment paradigm.

## Supplementary material

10.1136/rmdopen-2025-006180 online supplemental figure 1

10.1136/rmdopen-2025-006180 online supplemental file 1

## Data Availability

Data are available upon reasonable request.
