# Supplementary figures and images for: Efficacy and safety of iguratimod in patients with primary Sjögren’s syndrome: a multicentre randomised controlled trial
Source: RMD Open. 2025 Dec 19;11(4):e006180. doi: 10.1136/rmdopen-2025-006180 (PMC12718576; doi:10.1136/rmdopen-2025-006180)

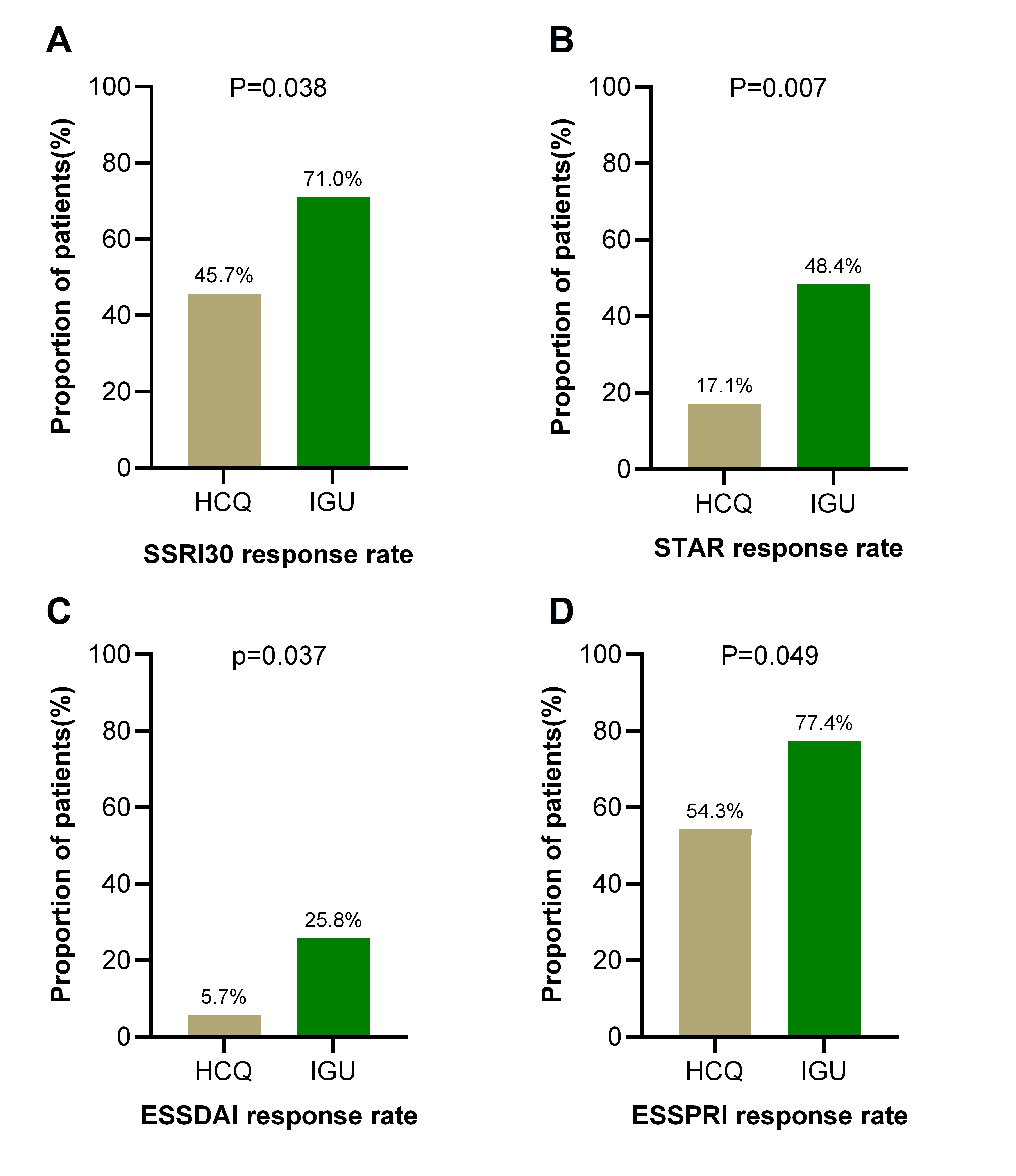

Supplement: online supplemental figure 1 [file rmdopen-11-4-s001.jpg]
