# Supplementary material for: Efficacy and safety of iguratimod in patients with primary Sjögren’s syndrome: a multicentre randomised controlled trial
Source: RMD Open. 2025 Dec 19;11(4):e006180. doi: 10.1136/rmdopen-2025-006180 (PMC12718576; doi:10.1136/rmdopen-2025-006180)
Supplement: online supplemental file 1 [file rmdopen-11-4-s002.docx]

**Efficacy and Safety of Iguratimod in the Treatment of Sjögren's Syndrome: A Multicenter Randomized Controlled Study**

**Study Protocol**

Lead Unit: The Second Affiliated Hospital Zhejiang University School of Medicine

Principal Investigator: Jing Xue

Responsible Department: Department of Rheumatology and Immunology

Contact Phone: 13858121751

Participating Units: The Second Affiliated Hospital Zhejiang University School of Medicine

Sir Run Run Shaw Hospital Zhejiang University School of Medicine

Jiaxing First Hospital

Zhuji People's Hospital

Changxing People's Hospital

Shaoxing Central Hospital

Study Duration: June 2021 – June 2023

Version Number: V 3.0

Version Date: December 17, 2021

Table of Contents

[I. Study Background 3](#_Toc23967)

[II. Study Objectives 4](#_Toc18644)

[III. Study Design Type, Principles, and Procedures 4](#_Toc11630)

[1.Study Design 4](#_Toc32609)

[2. Study Procedures 5](#_Toc29424)

[IV. Subject Selection 6](#_Toc25082)

[1. Inclusion Criteria 6](#_Toc26090)

[2. Exclusion Criteria 7](#_Toc7263)

[3. Elimination Criteria 7](#_Toc20962)

[4. Study Termination Criteria 7](#_Toc1951)

[V. Research Methods and Technical Route 8](#_Toc200)

[1. Study Drug Name and Specification 8](#_Toc11936)

[2. Treatment Plan 8](#_Toc24529)

[3. Concomitant Medications 8](#_Toc18432)

[VI. Observation Items and Testing Time Points 9](#_Toc22621)

[VII. Efficacy Evaluation Criteria 10](#_Toc22514)

[1. Primary Endpoint: 10](#_Toc25825)

[2. Secondary Endpoints: 10](#_Toc509)

[VIII. Safety Evaluation and Adverse Events 10](#_Toc27165)

[1. Safety Evaluation 10](#_Toc4559)

[2. Adverse Events 10](#_Toc24375)

[3. Serious Adverse Events 11](#_Toc15226)

[4. Unexpected Serious Adverse Drug Reactions 11](#_Toc25450)

[5. Evaluation of Causality for Adverse Events 11](#_Toc22238)

[6. Methods and Timing for Obtaining and Evaluating Adverse Events 12](#_Toc5737)

[7. Adverse Event Reporting Period 12](#_Toc14085)

[8. Adverse Event Recording Procedures 12](#_Toc28842)

[9. Death 12](#_Toc1468)

[IX. Quality Control and Quality Assurance of the Study 13](#_Toc818)

[1. Compliance 13](#_Toc3257)

[2. Standardized Operations 13](#_Toc668)

[3. Training 13](#_Toc24829)

[X. Data Safety Monitoring 13](#_Toc24427)

[XI. Statistical Analysis 14](#_Toc19042)

[1. Analysis Sets 14](#_Toc32236)

[2. Statistical Software 14](#_Toc20590)

[3. Statistical Methods 14](#_Toc29238)

[XII. Ethics of the Clinical Study 15](#_Toc24290)

[XIII. References 15](#_Toc2309)

I. Background

Primary Sjögren's Syndrome (pSS) is a chronic systemic autoimmune disease characterized by progressive infiltration of lymphocytes and plasma cells primarily targeting exocrine glands, leading to dysfunction of target organs. Clinically, it is mainly characterized by oral and ocular dryness, and can involve important organs such as the lungs, kidneys, and nervous system. pSS is a global disease, with a prevalence of 0.33% to 0.77% in the Chinese population, making it the most common autoimmune connective tissue disease in middle-aged and elderly adults, predominantly affecting women [1].

The exact pathogenesis of pSS is not fully understood but is generally believed to involve acquired immune abnormalities triggered by environmental factors such as viral infections on a genetic background, leading to the aggregation of lymphocytes and pro-inflammatory cytokines within glands, ultimately resulting in autoimmune glandular inflammation. Studies indicate [2-3] that B lymphocytes, alone or in cooperation with T lymphocytes, play a significant role in the pathogenesis of Sjögren's syndrome by causing abnormal proliferation and dysregulated apoptosis of B cells and the antibodies they produce, as well as the accumulation of hypergammaglobulinemia, in glands and organs. This manifests as various autoantibodies such as positive ANA, anti-SSA and anti-SSB antibodies, positive RF, hypergammaglobulinemia, increased B-cell ratio, elevated serum B-cell activating factor (BAFF) levels, and increased expression of BAFF receptors (BAFF-R) on target organs. Among these, hypergammaglobulinemia is a prominent manifestation of humoral immune abnormalities in pSS patients; immunoglobulins IgG, IgA, and IgM can all be elevated, but IgG elevation is most significant. Hypergammaglobulinemia in pSS often suggests that the disease may be in an active and progressive stage.

Treatment for pSS is divided into local and systemic therapy. The first-line treatment for oral and ocular dryness is symptom relief through local therapy. Patients with systemic involvement, especially active visceral organ involvement, may be treated with glucocorticoids, immunosuppressants, and biologics. Currently, the main goals of pSS treatment are to alleviate symptoms, halt disease progression, and prolong patient survival; there is no curative method.

Iguratimod (IGU) is a small molecule compound with anti-inflammatory and immunomodulatory effects. It is a Class I new drug approved by the China National Medical Products Administration (NMPA) and was launched in China in August 2011. It is currently indicated for the treatment of adult active rheumatoid arthritis (RA), effectively alleviating symptoms and reducing disease activity in RA patients with good tolerability and significantly better safety profile compared to the anchor drug methotrexate for RA. Iguratimod inhibits the production of various cytokines such as IFN-γ, IL-1, IL-6, TNF-α, IL-17, and immunoglobulins (IgM, IgG), significantly reducing inflammatory responses. Further research has found that Iguratimod can also inhibit B cells at different stages of differentiation, thereby suppressing excessive humoral immunity [4]. For connective tissue diseases whose pathological mechanism involves abnormal proliferation of large numbers of B cells and dysregulated apoptosis, these advantages suggest that Iguratimod may serve as a potential treatment. Multiple studies [5-12] have shown that Iguratimod has achieved certain efficacy in treating Sjögren's syndrome patients: it can effectively inhibit B-cell activation, reduce immunoglobulin levels, prevent multi-organ damage caused by hypergammaglobulinemia, and also reduce patients' disease activity scores (ESSDAI and ESSPRI scores). The Sjögren's Syndrome Group of the Rheumatology Branch of the Chinese Medical Doctor Association, in the "Diagnosis and Treatment Guidelines for Primary Sjögren's Syndrome" published in the Chinese Journal of Internal Medicine in 2020, also recommended the use of Iguratimod for treating Sjögren's syndrome [1]. Therefore, this study plans to enroll active pSS patients with hypergammaglobulinemia to evaluate the efficacy and safety of IGU monotherapy compared to Hydroxychloroquine (HCQ) monotherapy in pSS patients.

II. Study Objectives

Primary Objective: To evaluate the efficacy and safety of IGU monotherapy compared to Hydroxychloroquine (HCQ) monotherapy in pSS patients complicated with hypergammaglobulinemia.

III. Study Design Type, Principles, and Procedures

1.Study Design

This is a multicenter, open-label, randomized controlled clinical study. Eligible subjects will be randomly assigned in a 1:1 ratio to the experimental group (IGU monotherapy) and the control group (HCQ monotherapy) for 24 weeks of continuous dosing. The screening period is defined as the screening visit occurring within 4 weeks before the baseline visit. Subjects will undergo efficacy and safety assessments at weeks 0, 2, 6, 10, 16, and 24 after starting the study treatment (details see efficacy and safety endpoints) and are required to return to the hospital for follow-up within ±3 days of the corresponding follow-up points.

Sample Size Estimation: Using the SSRI-30 response rate at week 24 as the estimation indicator, based on references, the SSRI-30 response rate for HCQ treatment at week 24 is 17.6%. Assuming the SSRI-30 response rate for IGU treatment at week 24 is approximately 50% compared to the HCQ group, with a type I error rate of 5%, two-group comparison (1:1 allocation), two-sided test, and 80% power, 66 patients are estimated to be needed. Considering a 15% dropout rate, the sample size is increased to 78 patients.

Randomization: This trial will use a stratified randomization method, with a 1:1 ratio, and enrollment will be conducted by each center. The stratified randomization procedure will use a system provided by a third party. After screening each eligible subject, the researchers at each participating center will log into the randomization system, fill in the screening data, obtain the random number and corresponding drug number information, and dispense the corresponding study drug according to the random number and drug number.

The incidence of Sjögren's syndrome is relatively high, but treatment options are very limited, primarily relying on local replacement therapy to relieve dry eyes and mouth, with no effective drugs for systemic damage. B-cell overactivation is a hallmark of pSS, and IL-17 is positively correlated with the degree of lymphocyte infiltration in the labial glands of pSS patients. Furthermore, genome-wide association studies suggest that the NF-κB signaling pathway may also be involved in the pathogenesis of pSS. Iguratimod (IGU) is a novel DMARD approved in Japan and China for patients with rheumatoid arthritis (RA). Mechanistic studies indicate that IGU inhibits immunoglobulin production by B cells via the Protein Kinase C / Early Growth Response 1 (PKC/EGR1) pathway, while also inhibiting the IL-17 and NF-κB signaling pathways. Concurrently, some clinical data has accumulated for IGU in treating pSS, demonstrating that IGU can more effectively improve disease activity and various indicators in pSS patients.

2. Study Procedures


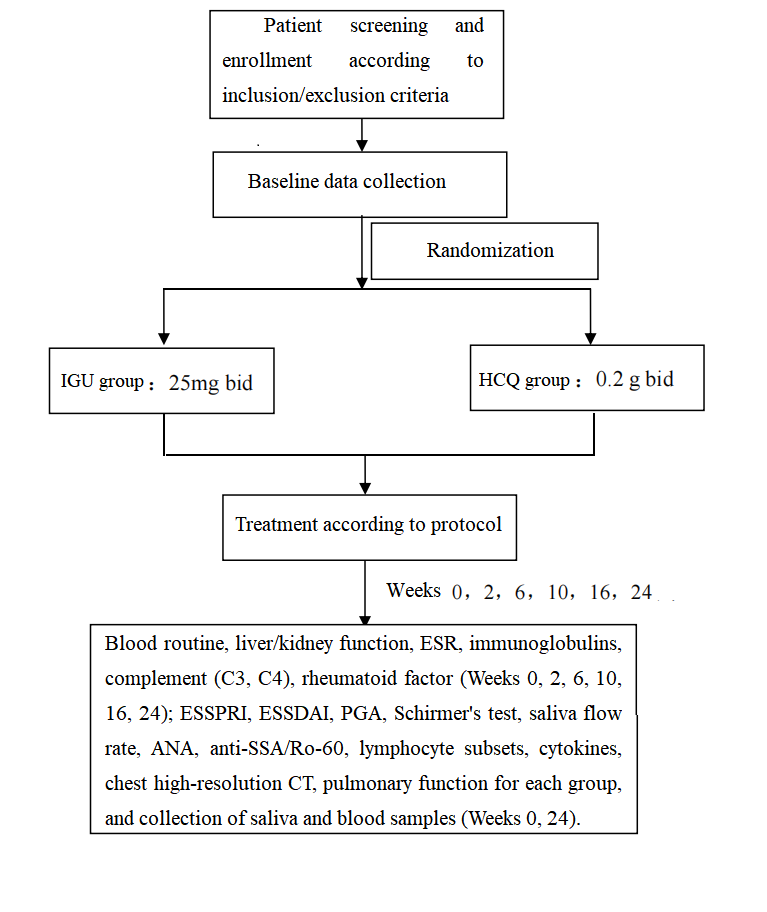


IV. Subject Selection

1. Inclusion Criteria

Subjects must meet all of the following criteria to be eligible:

1) Age 18 to 70 years (as of screening day), any gender;

2) Diagnosed with primary Sjögren's Syndrome according to the 2016 ACR/EULAR classification criteria;

3) Patient reports symptoms of dry mouth and/or dry eyes;

4) Patient is positive for autoantibodies (anti-SSA/Ro-60);

5) Patient has hyperglobulinemia (IgG ≥16 g/L);

6) No treatment with glucocorticoids, immunosuppressants, biologics, etc., within 4 weeks before screening;

7) For women of childbearing potential, pregnancy test is negative; agrees to use effective contraception during the trial;

8) Has not participated in any drug trial within 12 weeks prior to enrollment;

9) Understands the purpose and procedures of the trial and voluntarily signs the written informed consent form.

2. Exclusion Criteria

Subjects meeting any of the following criteria will be excluded:

1) Pregnant, lactating, or planning pregnancy in the near future, or unwilling to use reliable contraception during the study;

2) Combined with other connective tissue diseases;

3) Combined with malignant tumors;

4) Patients with psychiatric disorders, history of alcoholism, immunodeficiency, uncontrolled infections, or drug or other substance abuse;

5) Involvement of important organs requiring additional glucocorticoids, other immunosuppressants, or biologics to control the condition: heart, liver (transaminases/bilirubin >1.5 times ULN), kidneys (Cr ≥133 mmol/L), lungs (FVC % <60%), hematologic system (WBC <2.5×10⁹/L, HGB <80 g/L, PLT <80 ×10⁹/L), nervous system lesions, etc.;

6) Presence of fundus/visual field lesions;

7) Allergy to any component of the study drugs (IGU and/or HCQ);

8) Other conditions considered by the investigator as unsuitable for trial entry.

3. Elimination Criteria

1) Incorrectly enrolled patients, i.e., those who do not meet the inclusion criteria but are enrolled;

2) Patients who never used the trial drug after enrollment, or have no follow-up records, affecting efficacy and safety evaluation;

3) Violation of the protocol regarding concomitant medication;

4. Study Termination Criteria

1) Poor subject compliance, core drug usage outside 80%-120%;

2) Subject unwilling to continue participation or lost to follow-up;

3) Subject pregnancy;

4) Worsening of subject's condition or occurrence of serious adverse events, including but not limited to: drug allergy, WBC <2.0 ×10⁹/L, transaminases ≥2 times ULN, etc.;

5) Other reasons considered by the investigator as making the subject unsuitable to continue the clinical trial.

V. Research Methods and Technical Route

1. Study Drug Name and Specification

Investigational Drug: Aidexin® (Iguratimod Tablets): Manufactured by Simcere Pharmaceutical Group (Hainan) Co., Ltd., specification 25mg/tablet, actual batch number applies, shelf life 2 years. Store protected from light, sealed.

Control Drug: Plaquenil® (Hydroxychloroquine Sulfate Tablets): Manufactured by Shanghai Shangyao Zhongxi Pharmaceutical Co., Ltd., specification 0.1g/tablet, actual batch number applies, shelf life 3 years. Store sealed, below 25°C.

2. Treatment Plan

This is a randomized, controlled, multicenter clinical study. Eligible subjects will be randomly assigned in a 1:1 ratio to the following groups for 24 weeks of continuous dosing.

Experimental Group: Iguratimod group 25 mg, orally, one tablet, twice daily.

Control Group: Hydroxychloroquine group 0.1 g, orally, two tablets, twice daily.

3. Concomitant Medications

At each visit, the investigator shall accurately record the patient's concomitant medications. Includes medication name, purpose, start/stop time, method, and dose.

Drugs not allowed during the study: Except for the trial drugs, the use of glucocorticoids, other immunosuppressants or immunomodulators, biologics, Chinese patent medicines, etc., that may affect the evaluation of the disease's efficacy is prohibited during the observation period.

Drugs allowed during the study: Patients requiring long-term medication for underlying diseases such as diabetes, hypertension, etc., may continue taking them; Stable doses of calcium supplements, such as Vitamin D, calcium preparations, can continue to be used; Drugs for preventing or treating leukopenia and thrombocytopenia, such as Leucogen tablets, can be used concomitantly if judged necessary by the clinician; Drugs to alleviate symptoms such as liver-protecting or stomach-protecting agents can be used concomitantly if judged necessary by the clinician, and recorded.

VI. Observation Items and Testing Time Points

| Items | Week 0 | Week 2 | Week 6 | Week 10 | Week 16 | Week 24 |
| --- | --- | --- | --- | --- | --- | --- |
| Physical examination | √ | √ | √ | √ | √ | √ |
| ANA | √ |  |  |  |  | √ |
| ESSDAI | √ |  |  |  |  | √ |
| ESSPRI | √ |  |  |  |  | √ |
| Schirmer's test | √ |  |  |  |  | √ |
| USFR | √ |  |  |  |  | √ |
| Lymphocyte subsets | √ |  |  |  |  | √ |
| HRCT |  |  |  |  |  |  |
| Complete blood count | √ | √ | √ | √ | √ | √ |
| Urinalysis | √ | √ | √ | √ | √ | √ |
| Liver function | √ | √ | √ | √ | √ | √ |
| Kidney function | √ | √ | √ | √ | √ | √ |
| Immunoglobulins and complements | √ | √ | √ | √ | √ | √ |
| RF | √ | √ | √ | √ | √ | √ |
| ESR | √ | √ | √ | √ | √ | √ |
| CRP | √ | √ | √ | √ | √ | √ |

Notes:

1. Baseline HRCT can use imaging examinations within 6 months before week 0; ANA can use tests within 3 months before week 0.

2. Unstimulated Saliva Flow Rate Measurement Method: Measure between 8-9 AM. The patient should not have eaten within the last hour, and should not smoke, chew gum, drink coffee, or other beverages within 1 hour. Rinse mouth several times with pure water, rest for 5 minutes; sit upright and relaxed. Instruct the patient to minimize movement during collection, especially oral movements, avoid chewing, swallowing, and talking; should keep eyes open. Before starting collection, ask the patient to swallow/empty existing saliva in the mouth. Start timing saliva collection. The patient leans head forward, mouth slightly open, allowing saliva to flow directly into the cup; time for 15 minutes; after finishing, ask the patient to quickly spit residual saliva from the mouth into the cup. Measure saliva volume with a syringe, calculate unstimulated whole saliva flow rate (ml/min).

VII. Efficacy Evaluation Criteria

1. Primary Endpoint:

Evaluate the between-group difference in the SSRI-30 response rate at week 24, defined as improvement ≥30% in at least two of the following five items: fatigue, dry mouth, dry eyes based on patient VAS, unstimulated whole saliva flow rate, and erythrocyte sedimentation rate.

2. Secondary Endpoints:

1) Difference in ESSPRI at week 24 compared to baseline;

2) Difference in ESSDAI at week 24 compared to baseline;

3) Difference in Schirmer's test at week 24 compared to baseline;

4) Difference in USFR at week 24 compared to baseline;

5) Difference in lymphocyte subsets at week 24 compared to baseline;

6) Difference in immunoglobulin levels at weeks 10 and 24 compared to baseline;

7) Difference in ESR levels at weeks 10 and 24 compared to baseline;

8) Difference in RF at weeks 10 and 24 compared to baseline.

VIII. Safety Evaluation and Adverse Events

1. Safety Evaluation

Evaluate the safety of all subjects who received at least one dose of the study drug. Safety outcomes include adverse events, clinically significant vital sign changes, laboratory abnormalities, and clinical drug tolerance. The investigator shall determine whether each adverse event is mild, moderate, severe, or very severe.

2. Adverse Events

According to the International Council for Harmonisation (ICH), an Adverse Event (AE) is any untoward medical occurrence in a patient or clinical investigation subject administered a pharmaceutical product, regardless of causal relationship. Therefore, an AE can be any of the following:

- Any unfavorable and unintended sign (including an abnormal laboratory finding), symptom, or disease temporally associated with the use of a medicinal product, whether or not considered related to the medicinal product;

- Any new disease or exacerbation of an existing disease (worsening in nature, frequency, or severity under known conditions);

- Recurrence of an intermittent medical condition not present at baseline (e.g., headache);

- Worsening in any laboratory value or other clinical test, associated with symptoms or leading to changes in study treatment or concomitant therapy, or discontinuation of the study drug.

3. Serious Adverse Events

A Serious Adverse Event (SAE) is any AE that meets any of the following criteria:

- Fatal (i.e., the AE actually causes or leads to death);

- Life-threatening; (Note: "Life-threatening" means the patient was at immediate risk of death at the time of the event; it does not refer to an event that hypothetically might cause death if it were more severe.)

- Requires inpatient hospitalization or prolongation of existing hospitalization;

- Results in persistent or significant disability/incapacity (i.e., the AE results in severe disruption of the patient's normal life functions);

- Considered a significant medical event by the physician (e.g., may jeopardize the patient or may require medical/surgical intervention to prevent one of the outcomes listed above).

4. Unexpected Serious Adverse Drug Reactions

An adverse reaction whose nature, severity, outcome, or frequency is not consistent with the expected risk described in the prior protocol or other relevant materials.

5. Evaluation of Causality for Adverse Events

The physician should decide whether an AE is related to the study drug based on knowledge of the patient, the circumstances of the event, and any other potential causes, indicating "Yes" or "No". Consider the following:

1) Temporal relationship between event onset and start of study drug use;

2) The event's course, especially considering the effect of dose reduction or discontinuation of the study drug, or re-challenge (when applicable);

3) Known events associated with the study drug or similar treatments;

4) Known events associated with the disease under investigation;

5) Known risk factors present in the patient or use of concomitant medications that may increase the event's occurrence;

6) Presence of non-treatment-related factors known to be associated with the event;

7) For patients receiving concomitant therapy, causality will be assessed separately for each drug.

6. Methods and Timing for Obtaining and Evaluating Adverse Events

The physician is responsible for ensuring that all collected AEs are recorded in the appropriate forms according to the protocol and reported to the sponsor as per the protocol's instructions.

For each recorded AE, the physician will assess severity, seriousness, and causality.

7. Adverse Event Reporting Period

The physician will seek information about AEs during every contact with each patient. All AEs listed in the protocol that need to be collected and reported (whether reported by the patient or discovered by the research staff) will be recorded in the patient's medical record.

Once a patient is enrolled, AEs will be collected until the end of their observation period. After this period, the physician does not need to actively monitor for AEs, but if the treating physician becomes aware of any AE related to any drug, these events should be reported to the principal investigator.

8. Adverse Event Recording Procedures

When recording an AE, the physician should use precise medical terminology/concepts. Avoid using colloquial language or abbreviations.

9. Death

All events with a fatal outcome or result should be classified as Serious Adverse Events (SAEs). All deaths occurring during the protocol-defined AE reporting period, regardless of causality with the drug regimen, must be recorded in the AE form and reported immediately to the sponsor. Death should be considered an outcome, not a unique event. The event or condition that caused or led to the fatal outcome should be recorded as a separate medical concept. The term "sudden death" should only be used for patients with or without pre-existing heart disease, presumed due to cardiac causes, dying suddenly and unexpectedly within one hour of the onset of acute symptoms, or unwitnessed death occurring within 24 hours of the patient last being seen alive or in a stable condition. If the cause of death is unknown and cannot be determined at the time of reporting, "death of unknown cause" should be recorded on the AE report form. If the cause of death is later obtained (e.g., after autopsy), "death of unknown cause" should be replaced by the identified cause of death.

IX. Quality Control and Quality Assurance of the Study

1. Compliance

The trial must be implemented only after the study protocol has been approved by the Ethics Committee. Before obtaining EC review and written approval, the investigator shall not arbitrarily revise or change the study protocol, except to eliminate immediate risk to subjects. Any protocol deviations must be recorded in the CRF.

If protocol changes are made before EC approval to eliminate subject risk, a change application should be submitted to the EC as soon as possible.

2. Standardized Operations

Based on compliance with GCP (2020 edition) and drug registration regulations, the trial drugs are obtained within medical institutions. Laboratory tests are performed by each trial center according to Standard Operating Procedures (SOPs). The clinical laboratories of each trial center must perform internal quality control as required and obtain quality evaluation qualification certificates from the National Center for Clinical Laboratories.

3. Training

Before the clinical trial begins, the responsible person at each trial center should train the investigators on the trial protocol, ensure they read and understand the content of this clinical trial protocol, master GCP principles, unify recording methods and judgment standards, and strictly execute according to the protocol.

X. Data Safety Monitoring

The clinical study will develop corresponding data safety monitoring plans based on risk level. All adverse events will be recorded in detail, properly managed, and followed up until properly resolved or the condition stabilizes. Serious adverse events and unexpected events will be reported promptly to the Ethics Committee, regulatory authorities, the sponsor, and drug regulatory authorities as required; the principal investigator will periodically review all adverse events cumulatively, and investigator meetings will be convened if necessary to assess the study's risks and benefits; for blinded trials, emergency unblinding may be performed if necessary to ensure subject safety and rights; studies with greater than minimal risk will arrange for an independent data monitor to monitor the study data; high-risk studies will establish an independent Data Safety Monitoring Board (DSMB) to monitor cumulative safety and efficacy data to make recommendations on whether the study should continue.

XI. Statistical Analysis

1. Analysis Sets

1) The Full Analysis Set (FAS) includes almost all subjects who entered the study. Only subjects excluded during screening and not enrolled or enrolled but with no follow-up data are excluded from the FAS population.

2) The Per Protocol Set (PPS) is a subset of the FAS, consisting of subjects with good compliance and no major protocol violations. Generally, it refers to subjects in the FAS meeting the following three conditions: A) Complete baseline values for primary indicators; B) No protocol violations, meeting inclusion/exclusion criteria, no concomitant use of prohibited drugs; C) Good compliance.

3) The Safety Analysis Set (SS) includes all subjects who entered the study, received at least one dose of treatment, and had at least one safety assessment.

2. Statistical Software

Statistical analysis will be performed using SPSS software V22.0.

3. Statistical Methods

1) General Analysis: For continuous data meeting normality, use mean ± standard deviation for statistical description; if not met, use median (interquartile range). Categorical data use frequency (percentage) for statistical description. For between-group comparisons of continuous data, if normality and homogeneity of variance are satisfied, use t-test or ANOVA for difference analysis; if not, use non-parametric tests (e.g., Wilcoxon rank-sum test). For categorical data comparisons, use chi-square test or Fisher's exact test as appropriate. All tests are two-sided with a significance level α of 0.05.

2) Efficacy Analysis: Primarily based on the Full Analysis Set (FAS) and the Per Protocol Set (PPS). For the primary endpoint, use chi-square test or Fisher's exact test to compare the SSRI-30 response rate at week 24 between groups. For secondary and other endpoints, use the general analysis methods described above to test between-group differences; for longitudinal efficacy data, use mixed-effects models as appropriate. In the mixed-effects model, values at baseline and post-treatment will be the dependent variable, patient and center levels as random effects, group and follow-up time as independent variables, adding a group and follow-up time interaction term to estimate the difference between values at week 24 and baseline within each group.

3) Safety Analysis: Based on the Safty Set (SS). Safety analysis will primarily use descriptive statistics. Summarize AEs, Treatment-Emergent AEs (TEAEs), SAEs, laboratory data, vital signs, etc. If comparisons are needed, use chi-square test or Fisher's exact test for between-group analysis.

XII. Ethics of the Clinical Study

The clinical study will adhere to the World Medical Association's Declaration of Helsinki and other relevant regulations. Before the study begins, the protocol must be approved by the Ethics Committee. Before each subject is enrolled, the investigator is responsible for fully and comprehensively explaining the study's purpose, procedures, and potential risks to the subject or their representative, and obtaining signed written informed consent. Subjects should know they have the right to withdraw from the study at any time. Informed consent forms shall be retained as clinical study documents for reference. The study will protect subjects' personal privacy and data confidentiality.

XIII. References

[1] Zhang W, Li XM, Xu D, et al. Diagnosis and treatment guidelines for primary Sjögren's syndrome [J]. Chinese Journal of Internal Medicine, 2020, 59(4): 269-276.

[2] Kroese FG, Abdulahad WH, Haacke E, et al. B-cell hyperactivity in primary Sjögren's syndrome[J]. Expert Rev Clin Immunol, 2014, 10(4): 483-99.

[3] Lin DF, Zhang W, Zhao Y. The role of B cells in primary Sjögren's syndrome[J]. Chinese Journal of Clinical Immunology and Allergy, 2009, 3(3): 216-220.

[4] Ye Y, Liu M, Tang L, et al. Iguratimod represses B cell terminal differentiation linked with the inhibition of PKC/EGR1 axis[J]. Arthritis Res Ther, 2019, 21(1): 92.

[5] Wang X, Yuan X, Wang QK, et al. Therapeutic effect and mechanism of iguratimod on primary Sjögren's syndrome[J]. Chinese Journal of Disease Control & Prevention, 2018, 22(1): 75-78.

[6] Jiang W, Zhang L, Zhao Y, et al. The efficacy and mechanism for action of iguratimod in primary Sjögren's syndrome patients[J]. Int Ophthalmol, 2020, 40(11): 3059-3065.

[7] Ye Y, Liu M, Tang L, et al. Iguratimod represses B cell terminal differentiation linked with the inhibition of PKC/EGR1 axis[J]. Arthritis Res Ther, 2019, 21(1): 92.

[8] Jiang DX, Bai YJ, Zhao LP, et al. Clinical observation of iguratimod combination therapy for primary Sjögren's syndrome[J]. Clinical Misdiagnosis & Mistherapy, 2016, 29(8): 90-93.

[9] Xu D, Lu XW, Cui P, et al. Comparison of efficacy and safety of iguratimod versus hydroxychloroquine in the treatment of Sjögren's syndrome patients[J]. Journal of Difficult and Complicated Cases, 2017, 16(9): 915-918.

[10] Luo QW, Guo DM, Yu YT, et al. Efficacy and safety of iguratimod versus hydroxychloroquine in the treatment of Sjögren's syndrome patients[J]. Chinese Journal of Clinical Research, 2018, 10(24): 94-95.

[11] Li CJ, Li R, Liu HZ, et al. Efficacy of methylprednisolone combined with iguratimod in the treatment of primary Sjögren's syndrome and its effect on immunoglobulin levels[J]. China Pharmaceuticals, 2018, 27(14): 35-37.

[12] Chen H, Qi X, Li Y, et al. Iguratimod treatment reduces disease activity in early primary Sjögren's syndrome: An open-label pilot study[J]. Mod Rheumatol, 2020: 1-5.
